# Community-curated Galaxy interfaces with the Galaxy Labs Engine

**DOI:** 10.1093/gigascience/giag041

**Published:** 2026-04-08

**Authors:** Cameron J Hyde, Anna Syme, Bérénice Batut, Paul F Zierep, Winnie Mok, Wendi A Bacon, Gareth R Price

**Affiliations:** QCIF Data and Software Solutions, Level 1, 315 Brunswick Street, Fortitude Valley, Brisbane, 4006, Queensland, Australia; University of Melbourne, Grattan Street, Parkville, Victoria, 3010, Australia; Australian BioCommons, Faculty of Medicine, University of Melbourne, Grattan Street, Parkville, Victoria, 3010, Australia; IFB-core, Institut Français de Bioinformatique (IFB), CNRS, INSERM, INRAE, CEA, 94800 Villejuif, France; Plateforme AuBi, Mésocentre Clermont-Auvergne, Université Clermont Auvergne, 49, Boulevard François-Mitterrand, Aubière, France; Department of Computer Science, University of Freiburg, Georges-Koehler-Allee 079, D-79110 Freiburg, Germany; Australian BioCommons, Faculty of Medicine, University of Melbourne, Grattan Street, Parkville, Victoria, 3010, Australia; School of Life, Health & Chemical Sciences, The Open University, Walton Hall, Milton Keynes, MK7 6AA, United Kingdom; Australian BioCommons, Faculty of Medicine, University of Melbourne, Grattan Street, Parkville, Victoria, 3010, Australia; Data Science, Collaborative Research Platform, University of Queensland, Sir Fred Schonell Drive, Brisbane, Queensland, 4072, Australia

**Keywords:** analysis, community, data, workflows, resources, FAIR, Galaxy

## Abstract

The Galaxy platform is a globally distributed environment for data-intensive research, providing thousands of analysis tools across major public servers. However, this decentralized ecosystem presents usability challenges for both users and administrators, particularly in surfacing relevant tools and workflows for specific communities. To improve discoverability and support global collaboration, the Galaxy project has employed community-driven “Galaxy Flavours”—subdomains with curated content for defined research domains. While conceptually valuable, Flavours suffer from critical limitations: They are statically deployed, difficult to replicate across servers, and often provide inconsistent and unintuitive user interfaces. To address these challenges, we developed the Galaxy Labs Engine (GLE), a service that enables the creation of Galaxy Labs. This new paradigm enables globally synchronized, domain-specific entry points built from structured, reusable web content. GLE separates content from deployment, allowing communities to define a shared canonical representation of their domain, while enabling individual Galaxy servers to locally customize presentation. Labs are designed to guide users through curated tools, workflows, and training resources, and are aimed at researchers who are new to the analytical methods or technologies specific to the domain. The GLE provides a consistent, customizable, and community-driven interface layer for the Galaxy ecosystem. By fostering FAIR principles, labs offer a scalable improvement to Flavours and enhance Galaxy’s ability to support diverse research communities. GLE is open-source and currently deployed at https://labs.usegalaxy.org.au, with multiple Labs already supporting active user groups. This work strengthens Galaxy’s role as a collaborative platform for reproducible, user-centred science.

## Findings

### Introduction

The Galaxy platform has supported online scientific data analysis for over two decades, evolving into a globally distributed ecosystem of public and private services. Its success and increasing scope have led to a proliferation of analytical tools and workflows tailored to local, regional, and international needs. Today, the four major Galaxy servers (known as usegalaxy.* and including .au, .eu, .fr, and .org) each offer thousands of data analysis tools [1]. Since each Galaxy server caters to different research communities and initiatives, these toolsets can vary considerably between servers, and they typically represent only a subset of the full Galaxy ToolShed inventory [1, 2].

This diversity, while powerful, presents challenges. For Galaxy administrators, there is constant tension between tailoring services to local needs and aligning with broader initiatives such as the Galaxy Training Network (GTN) [3] and the Vertebrate Genomes Project (VGP) [4]. Moreover, Galaxy’s decentralization hinders the ability to recommend alternative usegalaxy.* servers with confidence that they will provide equivalent analytical capabilities. For end users, navigating vast tool inventories to find the right tools, workflows, or training materials can be daunting and makes for poor user experience. Galaxy users are typically faced with a monolithic conglomeration of thousands of tools, which can be a great obstacle in the fulfillment of their analytical journey.

Galaxy’s development has always been community-driven. Without the traditional commercial feedback loop (e.g., sales), it relies on user engagement and the formation of Special Interest Groups (SIGs) to guide development priorities [1]. Galaxy Europe [5] pioneered the concept of tailored entry points—branded as “Galaxy Flavours”—which are subdomains targeting specific research domains or communities [6–13]. A Flavour (also known simply as a “Subdomain”) is essentially a “skin” over an existing Galaxy server, presenting the same tool set and compute backend with a filtered tool panel and customized landing page that presents tools, workflows, and other relevant resources to a SIG. Galaxy Flavours share a common user identity and storage quota with the “base” Galaxy server, providing unified access within a single Galaxy instance. This has great potential for the user experience, as each Flavour provides a different lens through which users can view the Galaxy server and its resources. Crucially, Flavours have the potential to lower the barrier to entry for novice users—those who are either new to computational methods or unfamiliar with the research domain. By surfacing domain-specific, community-endorsed tools and resources in a structured and approachable way, Flavours help guide these users into complex analytical environments without overwhelming them.

While Galaxy Flavours can technically be replicated between Galaxy services, they are deployed statically. Updates made on Galaxy Europe must be manually propagated elsewhere, creating an unfortunate maintenance burden. For a SIG to be effectively supported globally, its Flavour must be manually recreated on each major server, a process that has hindered global adoption and international SIG collaboration. Furthermore, the prospect of manually creating these instances “from scratch” presents a major obstacle for community participation. A further drawback of Flavours is inconsistency in user interface (UI) design, since landing pages tended to be rendered from bespoke, hard-coded Markdown files. Though simple and easy to develop, this approach tends to result in a linear list of web content, making navigation difficult for users. A specific UI issue typical of Flavours is the dreaded “Galaxy-in-Galaxy” anomaly, which occurs when a link to the Galaxy host is present in the landing page. Since the Flavour’s landing page is embedded within the Galaxy website, clicking these links can result in a second Galaxy page opening inside the initial one—clearly a confusing and unintended user experience.

While Galaxy Flavours provides a basic mechanism for channelling defined SIG users, global adoption requires a more refined implementation that addresses the above issues.

### Solution

To address these limitations, we developed a system to evolve Galaxy Flavours into globally synchronized, collaboratively managed resources. Our solution, termed a “Galaxy Lab”, is built on a web service called the Galaxy Labs Engine (GLE) [14]. GLE dynamically generates landing pages from central repositories of curated, domain-specific content. Individual Galaxy servers can then apply local customizations (e.g., branding, support links) while drawing from a shared, authoritative source of truth. This enables consistent, up-to-date, globally accessible entry points that reflect local contexts without duplicating content or effort. For clarity we have defined the language that will be used to describe this service in Table 1.

**Table 1:** Galaxy Labs Engine terminology.

Galaxy Lab	A subdomain of a Galaxy server that shows the Galaxy service with a custom landing page and tool box (previously known as a “Flavour”)
GLE	Galaxy Labs Engine
Lab page	The custom landing page shown by a Galaxy Lab, rendered by the GLE
Lab content	A remote GitHub folder of documents that the Labs Engine uses to render a Lab page
Content root	A YAML file in the Lab content which defines a specific Lab page. Each Lab can have multiple content roots to render the Lab page differently for each Galaxy server.
Lab creator	The person who uses the Labs Engine to build Lab pages, typically a research community member or web developer
Lab user	The target end-user for a Lab, typically a researcher
SIG	Special Interest Group—research community members with a common interest, e.g., Genomics
Subdomain	An alternative domain to serve a Galaxy Lab, e.g., genome.usegalaxy.org.au

One of the primary goals of Galaxy Labs is enhanced FAIRness (Findable, Accessible, Interoperable, Reusable) of Galaxy resources, which can become difficult for users to navigate due to the sheer volume of available content. The Galaxy ToolShed, for instance, boasts some 9,360 Galaxy tools at the time of writing [15]. While such volume and diversity of tools is undeniably positive for Galaxy users, the accessibility of these tools remains a challenge, since users struggle to identify the tools that address their research problems. Galaxy Labs aim to guide users through the resources that are available to them, and serve as a “soft landing” for users that are new to the platform or research topic.

Galaxy Labs offer user-friendly, purpose-built entry points to Galaxy servers. They support both novice and expert users by presenting streamlined, interactive interfaces that expose relevant tools, workflows, and training material with minimal configuration. By enhancing usability and promoting reproducibility, Galaxy Labs strengthen Galaxy’s mission as an open, community-centred platform for data-intensive science.

The GLE was designed to streamline the creation of landing pages for Galaxy Labs. Four core tenets were defined to guide software architecture and design according to FAIR principles:

1. Findable—Galaxy Labs are open source and globally accessible (as defined by each usegalaxy.* hosting Labs). Lab content is hosted in open GitHub repositories.
2. Accessible—The service should be sufficiently intuitive and well-documented to allow a SIG or community to create a page without programming and web development knowledge, and without having to deploy additional services. The Engine should render a standardized template to provide a familiar and consistent user experience between Labs and Galaxy servers, which does not depend on the Labs creator’s web design skills.
3. Interoperable—Lab content can be rendered for review and collaboration through the Lab Engine, without the requirement to host the Lab on a live Galaxy server.
4. Reusable—The service should provide mechanisms for customization by the requesting Galaxy server, such that metadata defined in a Galaxy Lab can be reused across any number of Galaxy servers.

**Figure 1:**
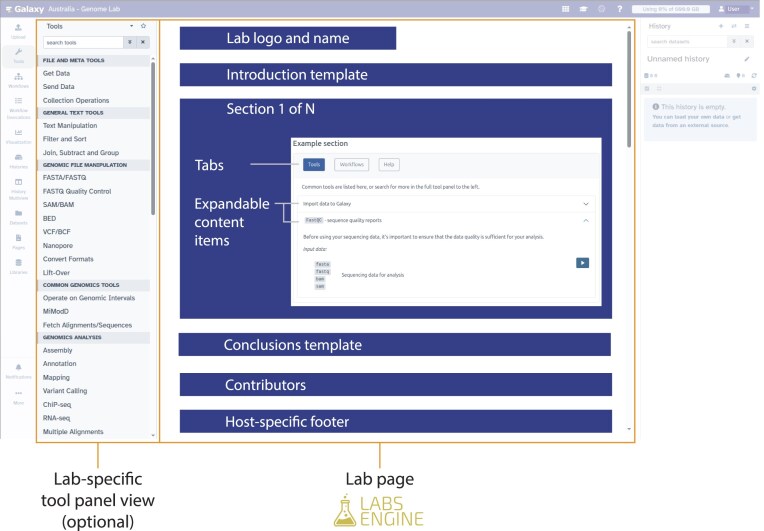
Annotated screenshot of a Galaxy Lab web interface, drawing attention to the components that are open to customization by Lab Creators. The central panel is rendered by the GLE and the Panel View on the left is an existing feature of the Galaxy framework.

The GLE is intended to give Galaxy communities the ability to easily build a Lab based on a framework of structured web content. This simplifies the process of building a UI that actively guides the user journey with layered content. When designing a Galaxy Lab with the Labs Engine, the Lab creator(s) should consider the diversity of user journeys that the Lab is intended to serve, and use that to structure the content. For example, where the users’ analysis tends to follow one of several data types or analytical methods, the Lab creator can use these to categorize and layer the Lab content by making use of Sections or Tabs (Fig. 1).

When including a particular resource (e.g., tool, workflow) in the Lab, care should be taken to ensure that the resource is sufficiently described in the Lab for a novice user to understand whether it is relevant to their analysis. This is fundamental to the user experience of a Galaxy Lab—the user should be able to navigate the page in such a way that they are guided towards the most appropriate resources Galaxy can offer, with as little friction as possible. Given a list of 10 tools, the user should not be expected to manually research each one to find out which is most appropriate—a Galaxy Labs should be designed to eliminate such friction. Whenever new content is added, the Lab Creators should ask themselves, “Does a novice user have enough information to know whether this is of use to them?” Conversely, the Lab creators must also be careful not to burden the user with too much information. For detailed documentation and instruction, they should consider linking to external content (such as tutorials from the GTN [3]) as frequently as required. In this way, the Lab can become a hub for Galaxy resources curated by the SIG.

Galaxy Labs are built by the communities who intend to use them, and can be deployed efficiently onto one or many global Galaxy services without the need for advanced programming experience of server access.

### Live examples of Galaxy Labs

The Genome Lab was the inaugural Galaxy Lab and served as a foundational canvas for developing the Lab concept and refining its UI. Its development was protracted but essential, allowing us to iteratively clarify the scope, structure, and purpose of what a “Galaxy Lab” should be. The design process was informed by direct engagement with the Threatened Species Initiative (TSI) [16], a nationally coordinated effort focused on supporting genomic analysis for Australian conservation biology. This use case underscored the need to accommodate a wide spectrum of user expertise, from novice researchers to experienced bioinformaticians. The Genome Lab’s development was also guided by Australian BioCommons community consultation, captured as roadmaps such as *Genome Assembly Infrastructure Roadmap for Australia* [17], which articulated the long-term vision for supporting domain-specific communities through reusable, federated infrastructure components like Labs.

To meet this need, our design went through multiple revisions of user experience design to shape an interface that was both approachable and functional. These design decisions helped define the Lab template now used across all Galaxy Labs. The Genome Lab now serves as a centralized, intuitive entry point for genome-scale analysis and training resources. Its content is dynamically generated from a curated repository [18], ensuring consistency and maintainability over time.

The Single-Cell Lab [19–21] evolved from multiple community-driven efforts to support single-cell transcriptomics in Galaxy, including the initial development of two separate analysis environments [10, 13]. These were later consolidated through the work of the Single-Cell and Spatial Omics Community (SPOC), a globally collaborative group focused on improving accessibility and reproducibility in single-cell analysis. This consolidation marked one of the earliest instances of a community-led engagement with the GLE, yielding valuable feedback on both process and interface design.

The Lab’s creation raised early challenges, including unclear governance of content and limited visual clarity in its initial presentation. Through iterative collaboration, SPOC helped shape a more maintainable and structured deployment model, informed by user-centred design principles. Notably, SPOC connected content streams from the GTN (such as FAQ, news, and event digests) into the Lab via streamlined, automated mechanisms. These integrations supported a dynamic, user-responsive environment while maintaining clarity and navigability. SPOC’s emphasis on workflow tagging and best practices reinforced the Lab’s goal of promoting reproducible science. Today, the Single-Cell Lab serves as a cohesive and navigable interface for users seeking community-driven, high-quality resources for single-cell and spatial omics analysis.

The Microbiology Lab [22] showcases the flexibility and scalability of the GLE by being deployed as a dedicated subdomain across multiple public Galaxy servers, including usegalaxy.eu, usegalaxy.org, usegalaxy.org.au, and usegalaxy.fr [23–26]. By offering a unified entry point to 300+ microbiology-specific tool suites, 100+ workflows, 35+ tutorials, and 15+ videos, the Lab enables streamlined access for researchers engaging in advanced microbial omics. It also exemplifies how the Labs Engine can be adapted for domain-specific use at scale, while maintaining a consistent user experience across instances.

The Microbiology Lab’s content is curated through the Galaxy CoDex GitHub repository [27, 28], which serves as the central hub for its interface components, tools, and workflows. Notably, the Lab features a hybrid content model: While some sections are manually designed for clarity and pedagogical value, two key areas—**Community-Curated Tools** and **Community Workflows**—are semi-automatically generated and regularly updated. This approach ensures the Lab remains both authoritative and dynamic, highlighting trusted, community-maintained resources and serving as a model for future Labs looking to balance manual editorial oversight with automation.

### Conclusion

We developed the GLE, a web service for generating and managing Galaxy Lab pages. GLE standardizes the structure and deployment of these entry points across Galaxy servers, enabling consistent and scalable delivery of domain-specific content. Crucially, it lowers the barrier for administrators and community members to create or modify Lab pages, broadening participation in their development.

Beyond facilitating local customization, GLE supports the rapid creation of exemplar Lab pages to demonstrate relevant tools and resources to new users. This promotes discoverability, onboarding, and engagement within specific research domains.

Now deployed to four major Galaxy servers around the globe, Galaxy Labs support a globally distributed model of collaborative development. With key Lab pages already deployed, researchers can access curated, context-appropriate tools, workflows, and training materials—enhancing usability, reproducibility, and community alignment across the Galaxy ecosystem.

## Methods

### Architecture and design

GLE is built on the Django web framework (version 5.1.4) [29], a library of the Python programming language (version 3.12) [30]. It integrates modern web technologies such as Bootstrap 5.1, FontAwesome, Material Icons, and jQuery 3.6, which empower Lab developers to design responsive and interactive interfaces. These libraries support advanced features, including web forms, embedded modals for supplementary content, and custom styling, all of which can be configured directly from within the community-actioned content repository. This modular architecture allows for granular control over page layout and behaviour without requiring changes to the core application. Effectively, this allows any public user to design their own Lab page and request it to be built on-the-fly through the Labs Engine website. A list of notable GLE features has been included in Table 2 for completeness.

**Table 2:** Feature catalogue for Galaxy Labs Engine.

Problem	Solution
Each Galaxy server must be able to customize each Lab page with local context.	Galaxy servers can specify a <myserver>.yml to override base configuration, enabling server-specific variables and content overrides.
Each Galaxy server must be able to customize the introductory text, footer and stylesheets to meet local requirements.	All Markdown snippets and static files can be customized by placing them in a <HOSTNAME> folder and specifying the custom path in <myserver>.yml.
Redundant content across different servers.	Introduces variable interpolation across YAML and Markdown/HTML to reuse content dynamically.
Lack of flexibility in content formatting.	Supports Markdown and HTML, with popular UI libraries available for use. Markdown “snippets” are injected in three locations in the page where the Lab developer has complete creative freedom.
Cluttered interfaces with lack of consistency between Labs.	All Labs are rendered from the same base template, which includes a standardized UI layout to ensure that Lab pages are intuitive and consistent. Dense content is structured into sections, tabs and collapsible elements.
Managing sections that are specific to certain Galaxy server(s).	Allows exclusion of specific items from certain hosts using the exclude_from directive the YAML content.
Requirement to host content on GitHub creates a slow development cycle.	Local rendering capability via a Python-based CLI to instantly preview changes before pushing to GitHub.
Delay in Lab page updates due to server-side caching.	Supports a cache=false flag to skip the caching. Local rendering bypasses cache entirely.
Capability for hosting static content (e.g., images) for use in Lab pages.	Images can be placed in the “static” folder of the content repository and then referenced in Markdown and YAML content.
Lab developers need constructive error messages when the expected Lab structure/schema is violated.	YAML content is validated and sanitized using the “pydantic” library. Error feedback is rendered into the requested web page for interpretation by the user.
A standardized, accessible mechanism for adding contributors.	The content repository can include a CONTRIBUTORS file which lists GitHub usernames of Lab contributors. These are rendered at the bottom of the Lab page.
Ability to embed content from other websites into Lab pages.	Since extended Markdown is supported, iframe elements can be used to embed content from any publicly visible website.
Links in the Lab page need to be screened to guarantee that the “Galaxy in Galaxy” anomaly does not occur.	All URLs in the rendered Lab page are programatically evaluated and configured to open in a new tab if they originate from the current web host.
Robust documentation for building new Lab pages.	The GLE landing page provides interactive documentation for building a Galaxy Lab page.
Landing on a new resource for the first time can be daunting for inexperienced users.	GLE accepts a video_url attribute in the <server>.yml metadata, which enables a YouTube video to be easily embedded in the Introduction of the Lab.

A notable aspect of the platform is its seamless integration with GitHub, which supports version-controlled content management and collaborative development practices. By hosting Lab page content in Git repositories, scientists benefit from transparent tests, simplified change rollbacks, and familiar collaboration workflows. This model aligns with open science principles and enhances reproducibility in computational research environments.

**Figure 2:**
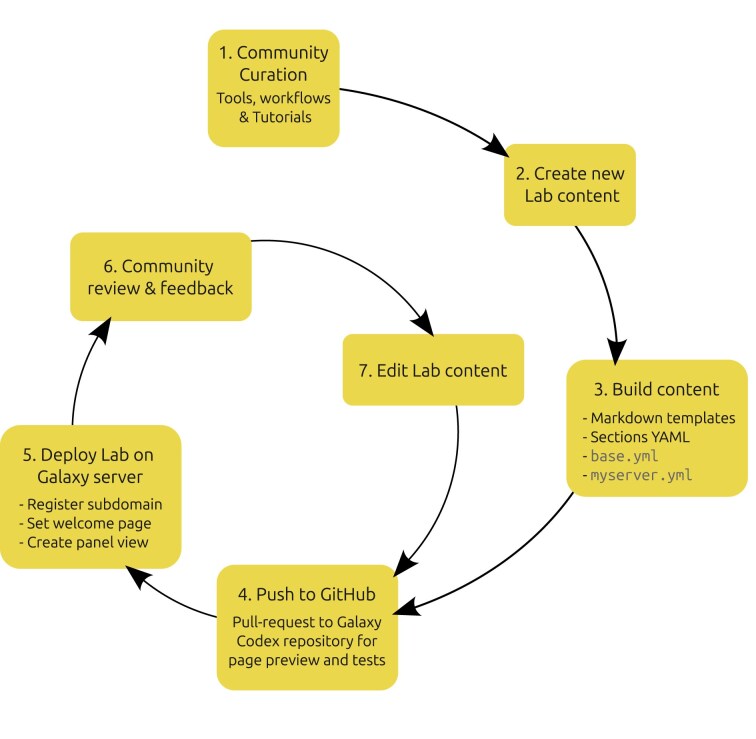
The lifecycle of web requests sent to the GLE. The left column shows actions that occur in the user’s web browser, the middle column shows processing that occurs on the Labs Engine server, and the right column shows content that is retrieved from the user’s GitHub repository.

**Figure 3:**
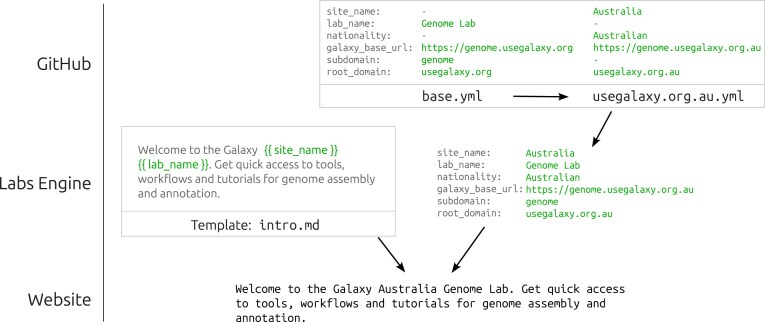
The diagram shows how variables defined in the requested <server>.yml file are interpolated to render locale-specific web content from Markdown or HTML templates. This feature allows one Lab to be rendered for multiple different locales by requesting different <server>.yml files.

Assuming that a prospective Lab creator has created the required files and uploaded them to a public GitHub repository, they can request the webpage to be built instantly at https://labs.usegalaxy.org.au/?content_root=GITHUB_URL, where GITHUB_URL is the URL of the root YAML file in their repository. The default root is the base.yml file, which tells the Labs Engine which other files and templates to include as well as setting variables such as site_name, which change how the Lab’s templates are rendered. The GLE request lifecycle is fully described in Fig. 2. Instead of requesting the base.yml root, Galaxy administrators should instead request their own <server>.yml root to return a webpage that is tailored to their server. For example, Galaxy Australia’s Labs uses the usegalaxy.org.au.yml root to display a page that has been customized with their local context. Figure 3 describes the data cascade that occurs on the GLE web server at the time of request to synthesize the requested content.

The GLE rendering mechanism is built around five core elements, where the first three are part of GLE and the last two are designed by a Lab creator and retrieved by GLE from a remote content repository:

1. **Base template**: The HTML base template from which all Lab pages are rendered.
2. **Section schema**: A schema that defines the accepted data structures for YAML content that populates the Sections component (Fig. 1). This is the main body of the Lab, which displays resources curated by the community with a structured UI.
3. **Rendering engine**: The web server endpoint that downloads YAML context, snippets, and other arbitrary content (from a location on GitHub); validates it against the Section schema; and renders it all into the base template to produce the Lab web page.
4. **YAML context**: A set of YAML files that provide the context for rendering templates and sections while also declaring which icon and snippets should be downloaded for the given Lab. Data defined in base.yml is overridden with a <server>.yml file in a cascade that provides fine-grained customization for each server that deploys a given Lab.
5. **Snippets**: Three Lab-specific Markdown templates (Introduction, Conclusion, and Footer), which are injected into specific locations in the base template to provide server-specific customization.

### Lab content structure

As described above, the “entrypoint” to the Lab content is the YAML context file, which sets variables and links to adjacent files in the content folder (Fig. 3). The content folder should also contain at least three folders—“templates”, “sections”, and “static”. The latter contains stylesheets and images to be used when rendering the Lab page. The “sections” folder contains a series of <section>.yml files that define structured content that will be rendered in the main body of the page (Fig. 1). The “templates” folder contains Markdown snippets that are used to render the Introduction, Conclusion, and Footer components of the Lab page (Fig. 1). These snippets provide the Lab creator a lot of flexibility for customizing the Lab for their analytical domain. First and foremost, the *Introduction* snippet provides an opportunity to address researchers in the Lab’s research domain directly, by explaining what this Lab is and why it could be useful for their research. The *Conclusion* snippet can be used for inserting any other web content that might be useful after the main content, such as an embedded news widget, links to external resources, or a request for feedback on the Galaxy Lab. Finally, the *Footer* snippet gives the Galaxy server an opportunity to display affiliations, funding sources and acknowledgement statements. Figure 3 provides a visual example of how cascading YAML context is injected into the “Introduction” Snippet to create layered customization for a Lab page.

### User interface design

One of the primary goals of the Labs Engine was to design a standardized UI that could deliver high content depth without overwhelming new users. The chosen design features a UI component that has been named a *Section*. These nested components allow large amounts of content to be structured concisely and clearly, such that the user can navigate the content quickly without being overwhelmed. In order of hierarchy, these components are (1) Section, (2) Tab, and (3) Item (Fig. 1). Importantly, a Lab can define as many of these components as required, depending on the amount of resources that have been curated by the community. The Section component (Fig. 1) is repeated vertically down the page, and divides the content into discrete primary categories. These categories should be defined at the discretion of the Lab creator, but a common pattern is for section categories to follow a typical analytical user journey—for example, *Data upload*, *Data QC*, *Analysis*, *Visualization*. Each Section contains one or more Tabs (Fig. 1), which function just like the tabs in a web browser, allowing the user to navigate sub-categories within the Section. Again, the structure of tabs is left to the discretion of the creators, but a “Data upload” Section might define tabs that follow a user journey such as *Getting started*, *Tools*, *Tutorials*, or perhaps appeal to a series of user personas such as *Datatype X*, *Datatype Y*, or *Datatype Z*. Finally, the content Items (Fig. 1) within each Tab contain a title and body (in Markdown format), an *input datatypes* list (a structured format to describe tools and workflows), and button links with configurable URL, tooltip, and text or icon. These items are rendered as a stack of expandable boxes (known as an “Accordion”), which allows the user to quickly scan the titles of available items, and then click to expand the full content. Importantly, the Markdown body can accommodate full HTML and be used to display generic web content such as bullet lists, images and even embedded widgets.

### Creating lab content

**Figure 4:**
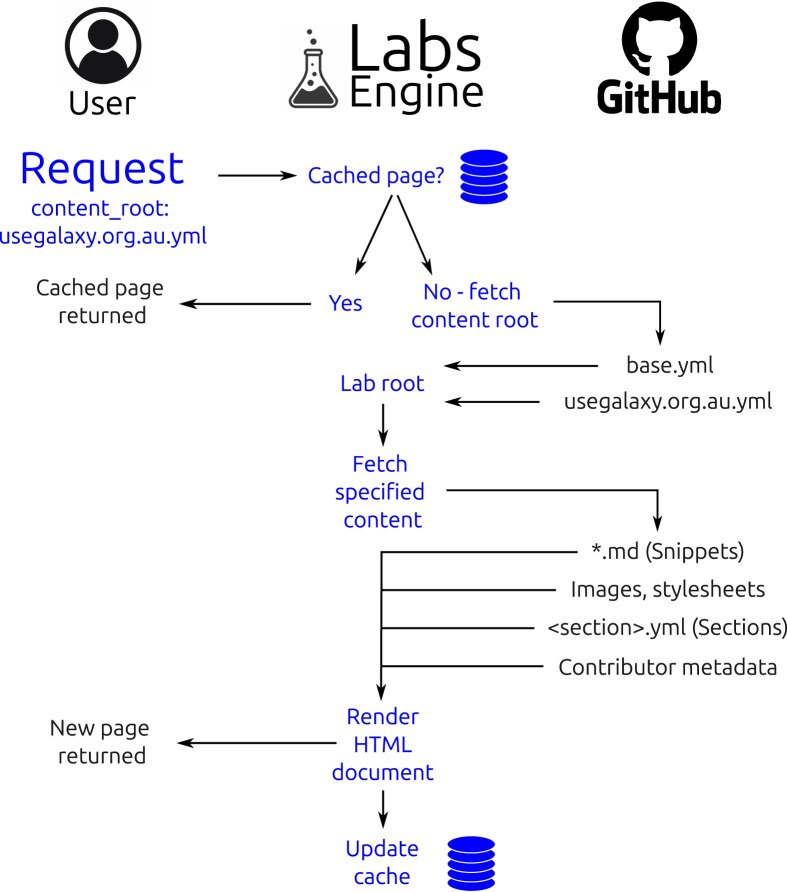
Development cycle for construction and maintenance of new Galaxy Lab. All steps aside from (5) can be carried out by the Galaxy community, without the assistance of the Galaxy server’s administrator, and step (5) requires such assistance only on initial deployment.

A central feature of GLE is that it supports rapid web page creation using preconfigured templates. The development lifecycle for generating a Galaxy Lab with GLE is described in Fig. 4. Community members can rapidly initiate a new Lab page by using a “boilerplate” generator [31], which generates a Zip archive of Lab content in the correct format and structure to get started. As the Lab creator edits the provided YAML and Markdown files, they can view their changes easily using the local rendering feature, which dramatically reduces the development feedback loop with real-time testing and refinement. When the creator is satisfied with their Lab content, all that is required to “deploy” the Lab page is to push it to an appropriate GitHub repository. Anyone can then view this page by making a request to GLE with their repository URL specified as the “content root” (e.g., https://labs.usegalaxy.org.au/?content_root=s://github.com/galaxyproject/galaxy_codex/blob/main/communities/genome/lab/base.yml).

Since a Lab page can take up to 20 seconds to render from scratch, GLE incorporates a caching mechanism to ensure fast load times. Rendered Lab pages are cached indefinitely, with a cache refresh being triggered by a GitHub workflow when changes are made to the content repository. This GitHub workflow is implemented in the “Galaxy Codex” repository [27, 28], the recommended location for globalized Lab content. This repository contains metadata, such as tools and workflows, that have been curated for different SIGs within the Galaxy community.

GLE aims to be self-documenting, with the landing page [14] itself being a Lab page that serves as a living example of GLE’s capabilities while also documenting the process of building and deploying a Lab page. GLE also includes a mock Lab page (*The Archaeology Lab*) [32] that creators can refer to for working examples of Lab content.

## Availability of supporting source code and requirements

Project name: galaxy-labs-engine

Project homepage: https://github.com/usegalaxy-au/galaxy-labs-engine

Operating system: Windows/Mac/Linux

Programming language: Python

Other requirements: NA

License: MIT license

## List of abbreviations

FAQ: frequently asked questions; GLE: Galaxy Labs Engine; GTN: Galaxy training network; HTML: HyperText Markup Language; QC: quality control; SIG: Special Interest Group; SPOC: Single-Cell and Spatial Omics Community; TSI: Threatened Species Initiative; UI: user interface; VGP: Vertebrate Genomes Project.

## Supplementary Material

giag041_Authors_Response_To_Reviewer_Comments_original_submission

giag041_GIGA-D-25-00301_original_submission

giag041_GIGA-D-25-00301_Revision_1

giag041_Reviewer_1_Report_original_submission Reviewer 1 -- 8/25/2025

giag041_Reviewer_2_Report_original_submission Reviewer 2 -- 2/3/2026

## Data Availability

An Ansible playbook for deploying a Labs Engine server can be found at GitHub repository [33]. Content repositories for Labs deployed by the Galaxy Project are open-source and can be found at GitHub repository [27]. Galaxy Labs Engine runs on a Docker stack which includes Nginx (latest version) and Gunicorn (version 22). It has been deployed on Linux Ubuntu 24.04 LTS, but is likely compatible with other Linux distributions.
