# Supplementary material for: Community-curated Galaxy interfaces with the Galaxy Labs Engine
Source: Gigascience. 2026 Apr 8;15:giag041. doi: 10.1093/gigascience/giag041 (PMC13148399; doi:10.1093/gigascience/giag041)
Supplement: giag041_GIGA-D-25-00301_original_submission [file giag041_giga-d-25-00301_original_submission.pdf]

GigaScience

Community-curated Galaxy interfaces with the Galaxy Labs Engine

--Manuscript Draft--

**Manuscript Number:**	GIGA-D-25-00301
**Full Title:**	Community-curated Galaxy interfaces with the Galaxy Labs Engine
**Article Type:**	Technical Note
**Funding Information:**	
**Abstract:**	The Galaxy platform is a globally distributed environment for data-intensive research, providing thousands of analysis tools across major public servers. However, this decentralised ecosystem presents usability challenges for both users and administrators, particularly in surfacing relevant tools and workflows for specific communities. To improve discoverability and support global collaboration, the Galaxy project has employed community-driven "Galaxy Flavours"—subdomains with curated content for defined research domains. While conceptually valuable, Flavours suffer from critical limitations: they are statically deployed, difficult to replicate across servers, and often provide inconsistent and unintuitive user interfaces.<br>To address these challenges, we developed the Galaxy Labs Engine (GLE), a service that enables the creation of Galaxy Labs. This new paradigm enables globally synchronised, domain-specific entry points built from structured, reusable web content. GLE separates content from deployment, allowing communities to define a shared canonical representation of their domain, while enabling individual Galaxy servers to locally customise presentation. Labs are designed to guide users through curated tools, workflows, and training resources, and are aimed at researchers who are new to the analytical methods or technologies specific to the domain.<br>The Galaxy Labs Engine provides a consistent, customisable, and community-driven interface layer for the Galaxy ecosystem. By fostering FAIR principles, Labs offer a scalable improvement to Flavours and enhance Galaxy's ability to support diverse research communities. GLE is open-source and currently deployed at https://labs.usegalaxy.org.au, with multiple Labs already supporting active user groups. This work strengthens Galaxy's role as a collaborative platform for reproducible, user-centered science.
**Corresponding Author:**	Wendi Bacon, PhD<br>The Open University<br>Milton Keynes, UNITED KINGDOM
**Corresponding Author Secondary Information:**	
**Corresponding Author's Institution:**	The Open University
**Corresponding Author's Secondary Institution:**	
**First Author:**	Cameron J Hyde
**First Author Secondary Information:**	
**Order of Authors:**	Cameron J Hyde
	Anna Syme
	Bérénice Batut
	Paul F Zierep
	Winnie Mok
	Wendi Bacon, PhD
	Gareth R Price
**Order of Authors Secondary Information:**	
**Additional Information:**	

Powered by Editorial Manager® and ProduXion Manager® from Aries Systems Corporation

Question	Response
Are you submitting this manuscript to a special series or article collection?	No
**Experimental design and statistics**<br><br>Full details of the experimental design and statistical methods used should be given in the Methods section, as detailed in our Minimum Standards Reporting Checklist. Information essential to interpreting the data presented should be made available in the figure legends.<br><br>Have you included all the information requested in your manuscript?	Yes
**Resources**<br><br>A description of all resources used, including antibodies, cell lines, animals and software tools, with enough information to allow them to be uniquely identified, should be included in the Methods section. Authors are strongly encouraged to cite Research Resource Identifiers (RRIDs) for antibodies, model organisms and tools, where possible.<br><br>Have you included the information requested as detailed in our Minimum Standards Reporting Checklist?	Yes
**Availability of data and materials**<br><br>All datasets and code on which the conclusions of the paper rely must be either included in your submission or deposited in publicly available repositories (where available and ethically appropriate), referencing such data using a unique identifier in the references and in the "Availability of Data and Materials" section of your manuscript.	Yes

Have you have met the above requirement as detailed in our Minimum Standards Reporting Checklist?	
GigaScience has policies and guidelines in place for the use of generative AI-writing tools such as ChatGPT. If you have used such writing tools to assist with writing the manuscript this must be declared and cited in the text. Authors should not list AI-writing tools and other AI-assisted technologies as an author or co-author and should acknowledge that they are fully responsible for text generated or refined by AI-writing tools.<p><br><br>A summary of use (particularly in the introduction or among methods) needs to be included at the end of the paper, and the outputs should also be included as a supplementary file hosted in GigaDB or other open repositories. Please read our guidelines for more information. <p><br><br>By submitting to GigaScience, you are aware of the journal's AI-writing tools policy, and if you have declared use of such tools below, you have acknowledged this where appropriate in your manuscript and have made a summary of use and outputs available. <p><br><b>AI-assisted writing tools have been used in the preparation of this manuscript?	Yes

Manuscript

Click here to access/download;Manuscript;Galaxy Labs - manuscript.docx

Community-curated Galaxy interfaces with the Galaxy Labs Engine

Cameron J Hyde[1], Anna Syme[2,3], Bérénice Batut[4,5], Paul F Zierep[6], Winnie Mok[3], Wendi A Bacon[7†] and Gareth R Price[3,8†]

† Indicates joint authorship

1. QCIF Data and Software Solutions, Brisbane, Queensland, Australia
2. University of Melbourne, Victoria, Australia
3. Australian BioCommons, Faculty of Medicine, University of Melbourne, Parkville, Victoria, Australia
4. IFB-core, Institut Français de Bioinformatique (IFB), CNRS, INSERM, INRAE, CEA, 94800 Villejuif, France
5. Plateforme AuBi, Mésocentre Clermont-Auvergne, Université Clermont Auvergne, Aubière, France
6. Department of Computer Science, University of Freiburg, Georges-Koehler-Allee 079, D-79110 Freiburg, Germany
7. School of Life, Health & Chemical Sciences, The Open University, Milton Keynes, United Kingdom
8. Data Science, Collaborative Research Platform, University of Queensland, Brisbane, Queensland, Australia

Abstract

The Galaxy platform is a globally distributed environment for data-intensive research, providing thousands of analysis tools across major public servers. However, this decentralised ecosystem presents usability challenges for both users and administrators, particularly in surfacing relevant tools and workflows for specific communities. To improve discoverability and support global collaboration, the Galaxy project has employed community-driven "Galaxy Flavours"—subdomains with curated content for defined research domains. While conceptually valuable, Flavours suffer from critical limitations: they are statically deployed, difficult to replicate across servers, and often provide inconsistent and unintuitive user interfaces.

To address these challenges, we developed the Galaxy Labs Engine (GLE), a service that enables the creation of Galaxy Labs. This new paradigm enables globally synchronised, domain-specific entry points built from structured, reusable web content. GLE separates content from deployment, allowing communities to define a shared canonical representation of their domain, while enabling individual Galaxy servers to locally customise presentation. Labs are designed to guide users through curated tools, workflows, and training resources, and are aimed at researchers who are new to the analytical methods or technologies specific to the domain.

The Galaxy Labs Engine provides a consistent, customisable, and community-driven interface layer for the Galaxy ecosystem. By fostering FAIR principles, Labs offer a scalable improvement to Flavours and enhance Galaxy's ability to support diverse research communities. GLE is open-source and currently deployed at https://labs.usegalaxy.org.au, with multiple Labs already supporting active user groups. This work strengthens Galaxy's role as a collaborative platform for reproducible, user-centered science.

Keywords

Analysis, community, data, workflows, resources, FAIR, Galaxy

Findings

Introduction

The Galaxy platform has supported online scientific data analysis for over two decades, evolving into a globally distributed ecosystem of public and private services. Its success and increasing scope have led to a proliferation of analytical tools and workflows tailored to local, regional, and international needs. Today, the four major Galaxy servers (known as usegalaxy.* and including .au, .eu, .fr, and .org) each offer thousands of data analysis tools [1]. Since each Galaxy server caters to different research communities and initiatives, these toolsets can vary considerably between servers, and they typically represent only a subset of the full Galaxy Toolshed inventory (https://toolshed.g2.bx.psu.edu) [1].

This diversity, while powerful, presents challenges. For Galaxy administrators, there is constant tension between tailoring services to local needs and aligning with broader initiatives such as the Galaxy Training Network (GTN) [2] and the Vertebrate Genomes Project (VGP) [3]. Moreover, Galaxy's decentralisation hinders the ability to recommend alternative usegalaxy.* servers with confidence that they will provide equivalent analytical capabilities. For end users, navigating vast tool inventories to find the right tools, workflows, or training materials can be daunting and makes for poor user experience. Galaxy users are typically faced with a monolithic conglomeration of thousands of tools, which can be a great obstacle in the fulfillment of their analytical journey.

Galaxy's development has always been community-driven. Without the traditional commercial feedback loop (e.g. sales), it relies on user engagement and the formation of Special Interest Groups (SIGs) to guide development priorities [1]. Galaxy Europe (usegalaxy.eu) pioneered the concept of tailored entry points—branded as "Galaxy Flavours"—which are subdomains targeting specific research domains or communities (https://usegalaxy-eu.github.io/posts/2020/12/28/subdomains/) [4-9]. A Flavour (also known simply as a "Subdomain") is essentially a "skin" over an existing Galaxy server, presenting the same tool set and compute backend with a filtered tool panel and customized landing page which presents tools, workflows and other relevant resources to a SIG. Galaxy Flavours share a common user identity and storage quota with the "base" Galaxy server, providing unified access within a single Galaxy instance. This has great potential for the user experience, as each Flavour provides a different lens through which users can view the Galaxy server and its resources. Crucially, Flavours have the potential to lower the barrier to entry for novice users - those who are either new to computational methods or unfamiliar with the research domain. By surfacing domain-specific, community-endorsed tools and resources in a structured and approachable way, Flavours help guide these users into complex analytical environments without overwhelming them.

While Galaxy Flavours can technically be replicated between Galaxy services, they are deployed statically. Updates made on Galaxy Europe must be manually propagated elsewhere, creating an unfortunate maintenance burden. For a SIG to be effectively supported globally, its Flavour must be manually recreated on each major server, a process which has hindered global adoption and international SIG collaboration. Furthermore, the prospect of manually creating these instances "from scratch" presents a major obstacle for community participation. A further drawback of Flavours is inconsistency in user interface design, since landing pages tended to be rendered from bespoke, hard-coded Markdown files. Though simple and easy to develop, this approach tends to result in a linear list of web content, making navigation difficult for users. A specific UI issue typical of Flavours is the dreaded "Galaxy-in-Galaxy" anomaly, which occurs when a link to the Galaxy host is present in the landing page. Since the Flavour's landing page is embedded within the Galaxy website, clicking these links can result in a second Galaxy page opening inside the initial one - clearly a confusing and unintended user experience.

While Galaxy Flavours provide a basic mechanism for channelling defined SIG users, global adoption requires a more refined implementation which addresses the above issues.

Solution

To address these limitations, we developed a system to evolve Galaxy Flavours into globally synchronised, collaboratively managed resources. Our solution, termed a “Galaxy Lab”, is built on a web service called the Galaxy Labs Engine (GLE), available at https://labs.usegalaxy.org.au. GLE dynamically generates landing pages from central repositories of curated, domain-specific content. Individual Galaxy servers can then apply local customisations (e.g. branding, support links) while drawing from a shared, authoritative source of truth. This enables consistent, up-to-date, globally accessible entry points that reflect local contexts without duplicating content or effort. For clarity we have defined the language that will be used to describe this service in Table 1.

Table 1: Galaxy Labs Engine terminology

Galaxy Lab	A subdomain of a Galaxy server which shows the Galaxy service with a custom landing page and tool box (previously known as a “Flavour”)
GLE	Galaxy Labs Engine
Lab page	The custom landing page shown by a Galaxy Lab, rendered by the Galaxy Labs Engine
Lab content	A remote GitHub folder of documents that the Labs Engine uses to render a Lab page
Content root	A YAML file in the Lab content which defines a specific Lab page. Each Lab can have multiple content roots to render the Lab page differently for each Galaxy server.
Lab creator	The person who uses the Labs Engine to build Lab pages, typically a research community member or web developer
Lab user	The target end-user for a Lab, typically a researcher
SIG	Special Interest Group - research community members with a common interest e.g. Genomics
Subdomain	An alternative domain to serve a Galaxy Lab, e.g. genome.usegalaxy.org.au

Galaxy Labs offer user-friendly, purpose-built entry points to Galaxy servers. They support both novice and expert users by presenting streamlined, interactive interfaces that expose relevant tools, workflows, and training material with minimal configuration. By enhancing usability and promoting reproducibility, Galaxy Labs strengthen Galaxy’s mission as an open, community-centred platform for data-intensive science.

The Galaxy Labs Engine was designed to streamline the creation of landing pages for Galaxy Labs. Four core tenants were defined to guide software architecture and design:

1. Web pages and their content should be open source and globally accessible.

2. The service should be sufficiently intuitive and well-documented to allow a SIG to create a page without programming and web development knowledge, and without having to deploy additional services.
3. The Engine should render a standardised template to provide a familiar and consistent user experience between Labs and between Galaxy servers.
4. The Engine should provide mechanisms for customization by the requesting Galaxy server.

The Galaxy Labs Engine is intended to give Galaxy communities the ability to easily build a Lab based on a framework of structured web content. This simplifies the process of building a user interface which actively guides the user journey with layered content. When designing a Galaxy Lab with the Labs Engine, the Lab Creator(s) should consider the diversity of user journeys that the Lab is intended to serve, and use that to structure the content. For example, where the users' analysis tends to follow one of several data types or analytical methods, the Lab Creator can use these to categorise and layer the Lab content by making use of Sections or Tabs (Fig. 1).

When including a particular resource (e.g. tool, workflow) in the Lab, care should be taken to ensure that the resource is sufficiently described in the Lab for a novice user to understand whether it is relevant to their analysis. This is fundamental to the user experience of a Galaxy Lab - the user should be able to navigate the page in such a way that they are guided towards the most appropriate resources Galaxy can offer, with as little friction as possible. Given a list of 10 tools, the user should not be expected to manually research each one to find out which is most appropriate - a Galaxy Labs should be designed to eliminate such friction. Whenever new content is added, the Lab Creators should ask themselves, "Does a novice user have enough information to know whether this is of use to them?". Conversely, the Lab Creators must also be careful not to burden the user with too much information. For detailed documentation and instruction, they should consider linking to external content (such as tutorials from the Galaxy Training Network [2]) as frequently as required. In this way, the Lab can become a hub for Galaxy resources curated by the SIG.

Galaxy Labs are built by the communities who intend to use them, and can be deployed efficiently onto one or many global Galaxy services without the need for advanced programming experience of server access.

Live examples of Galaxy Labs

The Genome Lab was the inaugural Galaxy Lab and served as a foundational canvas for developing the Lab concept and refining its user interface. Its development was protracted but essential, allowing us to iteratively clarify the scope, structure, and purpose of what a "Galaxy Lab" should be. The design process was informed by direct engagement with the Threatened Species Initiative (TSI) [15], a nationally coordinated effort focused on supporting genomic analysis for Australian conservation biology. This use case underscored the need to accommodate a wide spectrum of user expertise, from novice researchers to experienced bioinformaticians. The Genome Lab's development was also guided by Australian BioCommons community consultation, captured as roadmaps such as *Genome Assembly Infrastructure Roadmap for Australia* [16] , which articulated the long-term vision

for supporting domain-specific communities through reusable, federated infrastructure components like Labs.

To meet this need, our design went through multiple revisions of user experience design to shape an interface that was both approachable and functional. These design decisions helped define the Lab template now used across all Galaxy Labs. The Genome Lab now serves as a centralised, intuitive entry point for genome-scale analysis and training resources. Its content is dynamically generated from a curated repository [17], ensuring consistency and maintainability over time.

The Single-cell Lab [18, 19, 20] evolved from multiple community-driven efforts to support single-cell transcriptomics in Galaxy, including the initial development of two separate analysis environments [10, 11]. These were later consolidated through the work of the Single-cell and Spatial Omics Community (SPOC), a globally collaborative group focused on improving accessibility and reproducibility in single-cell analysis. This consolidation marked one of the earliest instances of a community-led engagement with the Galaxy Labs Engine, yielding valuable feedback on both process and interface design.

The Lab's creation raised early challenges, including unclear governance of content and limited visual clarity in its initial presentation. Through iterative collaboration, SPOC helped shape a more maintainable and structured deployment model, informed by user-centred design principles. Notably, SPOC connected content streams from the Galaxy Training Network (such as FAQ, News, and Event digests) into the Lab via streamlined, automated mechanisms. These integrations supported a dynamic, user-responsive environment while maintaining clarity and navigability. SPOC's emphasis on workflow tagging and best practices reinforced the Lab's goal of promoting reproducible science. Today, the Single-cell Lab serves as a cohesive and navigable interface for users seeking community-driven, high-quality resources for single-cell and spatial omics analysis.

The Microbiology Lab showcases the flexibility and scalability of the Galaxy Labs Engine by being deployed as a dedicated subdomain across multiple public Galaxy servers, including usegalaxy.eu, usegalaxy.org, usegalaxy.org.au, and usegalaxy.fr [21, 22, 23, 24]. By offering a unified entry point to 300+ microbiology-specific tool suites, 100+ workflows, 35+ tutorials and 15+ videos, the Lab enables streamlined access for researchers engaging in advanced microbial omics. It also exemplifies how the Labs Engine can be adapted for domain-specific use at scale, while maintaining a consistent user experience across instances.

The Microbiology Lab's content is curated through the Galaxy CoDex GitHub repository, which serves as the central hub for its interface components, tools, and workflows. Notably, the Lab features a hybrid content model: while some sections are manually designed for clarity and pedagogical value, two key areas—**Community-curated Tools** and **Community Workflows**—are semi-automatically generated and regularly updated. This approach ensures the Lab remains both authoritative and dynamic, highlighting trusted, community-maintained resources and serving as a model for future Labs looking to balance manual editorial oversight with automation.

Conclusion

We developed the Galaxy Labs Engine (GLE), a web service for generating and managing Galaxy Lab pages. GLE standardises the structure and deployment of these entry points across Galaxy servers, enabling consistent and scalable delivery of domain-specific content. Crucially, it lowers the barrier for administrators and community members to create or modify Lab pages, broadening participation in their development.

Beyond facilitating local customisation, GLE supports the rapid creation of exemplar Lab pages to demonstrate relevant tools and resources to new users. This promotes discoverability, onboarding, and engagement within specific research domains.

Now deployed to four major Galaxy servers around the globe, Galaxy Labs support a globally distributed model of collaborative development. With key Lab pages already deployed, researchers can access curated, context-appropriate tools, workflows, and training materials - enhancing usability, reproducibility, and community alignment across the Galaxy ecosystem.

Methods

Architecture and design

GLE is built on the Django web framework (version 5.1.4) [12], a library of the Python programming language (version 3.12) [13]. It integrates modern web technologies such as Bootstrap 5.1, FontAwesome, Material Icons, and jQuery 3.6, which empower Lab developers to design responsive and interactive interfaces. These libraries support advanced features including web forms, embedded modals for supplementary content, and custom styling, all of which can be configured directly from within the community-actioned content repository. This modular architecture allows for granular control over page layout and behavior without requiring changes to the core application. Effectively, this allows any public user to design their own Lab page and request it to be built on-the-fly through the Labs Engine website. A list of notable GLE features has been included in Table 2 for completeness.

[**Table 2**]

A notable aspect of the platform is its seamless integration with GitHub, which supports version-controlled content management and collaborative development practices. By hosting Lab page content in Git repositories, scientists benefit from transparent tests, simplified change rollbacks, and familiar collaboration workflows. This model aligns with open science principles and enhances reproducibility in computational research environments.

Assuming that a prospective Lab creator has created the required files and uploaded them to a public GitHub repository, they can request the webpage to be built instantly at `https://labs.usegalaxy.org.au/?content_root=GITHUB_URL`, where `GITHUB_URL` is the URL of the root YAML file in their repository. The default root is the `base.yml` file, which tells the Labs Engine which other files and templates to include as well as setting variables such as `site_name`, which change how the Lab's templates are rendered. The GLE request lifecycle is fully described in Figure 2. Instead of requesting the `base.yml` root, Galaxy

administrators should instead request their own `<server>.yml` root to return a webpage that is tailored to their server. For example, Galaxy Australia's Labs uses the `usegalaxy.org.au.yml` root to display a page which has been customized with their local context. Figure 3 describes the data cascade that occurs on the GLE web server at the time of request to synthesize the requested content.

The GLE rendering mechanism is built around five core elements, where the first three are part of GLE, and the last two are designed by a Lab creator and retrieved by GLE from a remote content repository:

1. **Base template**: the HTML base template from which all Lab pages are rendered.
2. **Section schema**: a schema which defines the accepted data structures for YAML content which populates the Sections component (Fig. 1). This is the main body of the Lab which displays resources curated by the community with a structured user interface.
3. **Rendering engine**: the web server endpoint which downloads YAML context, snippets and other arbitrary content (from a location on GitHub), validates it against the Section schema, and renders it all into the base template to produce the Lab web page.
4. **YAML context**: A set of YAML files which provide the context for rendering templates and sections, while also declaring which icon and Snippets should be downloaded for the given Lab. Data defined in `base.yml` is overridden with a `<server>.yml` file in a cascade that provides fine-grained customization for each server that deploys a given Lab.
5. **Snippets**: three Lab-specific Markdown templates (Introduction, Conclusion, Footer) which are injected into specific locations in the base template to provide server-specific customization.

Lab content structure

As described above, the "entrypoint" to the Lab content is the YAML context file, which sets variables and links to adjacent files in the content folder (Fig. 3). The content folder should also contain at least three folders - "templates", "sections" and "static". The latter contains stylesheets and images to be used when rendering the Lab page. The "sections" folder contains a series of `<section>.yml` files which define structured content that will be rendered in the main body of the page (Fig. 1). The "templates" folder contains Markdown Snippets that are used to render the Introduction, Conclusion and Footer components of the Lab page (Fig. 1). These snippets provide the Lab creator a lot of flexibility for customizing the Lab for their analytical domain. First and foremost, the *Introduction* snippet provides an opportunity to address researchers in the Lab's research domain directly, by explaining what this Lab is and why it could be useful for their research. The *Conclusion* snippet can be used for inserting any other web content that might be useful after the main content, such as an embedded news widget, links to external resources, or a request for feedback on the Galaxy Lab. Finally, the *Footer* snippet gives the Galaxy server an opportunity to display affiliations, funding sources and acknowledgement statements. Figure 3 provides a visual example of how cascading YAML context is injected into the "Introduction" Snippet to create layered customization for a Lab page.

User interface design

One of the primary goals of the Labs Engine was to design a standardized user interface (UI) that can deliver high content depth without overwhelming new users. The chosen design features a UI component that has been named a *Section*. These nested components allow large amounts of content to be structured concisely and clearly, such that the user can navigate the content quickly without being overwhelmed. In order of hierarchy, these components are 1) Section, 2) Tab, and 3) Item (Fig. 1). Importantly, a Lab can define as many of these components as required, depending on the amount of resources that have been curated by the community. The Section component (Fig. 1) is repeated vertically down the page, and divides the content into discrete primary categories. These categories should be defined at the discretion of the Lab creator, but a common pattern is for section categories to follow a typical analytical user journey - for example, "*Data upload*", "*Data QC*", "*Analysis*", "*Visualization*". Each Section contains one or more Tabs (Fig. 1), which function just like the tabs in a web browser, allowing the user to navigate sub-categories within the Section. Again, the structure of tabs is left to the discretion of the creators, but for a "Data upload" Section might define tabs that follow a user journey such as "*Getting started*", "*Tools*", "*Tutorials*", or perhaps appeal to a series of user personas such as "*Datatype X*", "*Datatype Y*", "*Datatype Z*". Finally, the content Items (Fig. 1) within each Tab contain a title and body (in Markdown format), an *input datatypes* list (a structured format to describe tools and workflows) and button links with configurable URL, tooltip and text or icon. These items are rendered as a stack of expandable boxes (known as an "Accordion") which allows the user to quickly scan the titles of available items, and then click to expand the full content. Importantly, the Markdown body can accommodate full HTML and be used to display generic web content such as bullet lists, images and even embedded widgets.

Creating Lab content

A central feature of GLE is that it supports rapid web page creation using preconfigured templates. The development lifecycle for generating a Galaxy Lab with GLE is described in Figure 2. Community members can rapidly initiate a new Lab page by using a "boilerplate" generator (https://labs.usegalaxy.org.au/bootstrap), which generates a Zip archive of Lab content in the correct format and structure to get started. As the Lab creator edits the provided YAML and Markdown files, they can view their changes easily using the local rendering feature, which dramatically reduces the development feedback loop with real-time testing and refinement. When the creator is satisfied with their Lab content, all that is required to "deploy" the Lab page is to push it to an appropriate GitHub repository. Anyone can then view this page by making a request to GLE with their repository URL specified as the "content root" (e.g. https://labs.usegalaxy.org.au/?content_root=https://github.com/galaxyproject/galaxy_codex/blob/main/communities/genome/lab/base.yml).

Since a Lab page can take up to 20 seconds to render from scratch, GLE incorporates a caching mechanism to ensure fast load times. Rendered Lab pages are cached indefinitely, with a cache refresh being triggered by a GitHub workflow when changes are made to the content repository. This GitHub workflow is implemented in the "Galaxy Codex" repository (https://github.com/galaxyproject/galaxy_codex) [14], the recommended location for

globalized Lab content. This repository contains metadata, such as tools and workflows, that have been curated for different SIGs within the Galaxy community.

GLE aims to be self-documenting, with the landing page (https://labs.usegalaxy.org.au) itself being a Lab page which serves as a living example of GLE's capabilities, while also documenting the process of building and deploying a Lab page. GLE also includes a mock Lab page (*The Archaeology Lab;* https://labs.usegalaxy.org.au/?content_root=https://raw.githubusercontent.com/usegalaxy-au/galaxy-labs-engine/refs/heads/main/app/labs/static/labs/content/simple/base.yml) that creators can refer to for working examples of Lab content.

Availability of supporting source code and requirements

Galaxy Labs Engine is open-source and available at https://github.com/usegalaxy-au/galaxy-labs-engine. An Ansible playbook for deploying a Labs Engine server can be found at https://github.com/usegalaxy-au/infrastructure/blob/master/galaxy-labs_engine_playbook.yml. Content repositories for Labs deployed by the Galaxy Project are open-source and can be found at https://github.com/galaxyproject/galaxy_codex.

Galaxy Labs Engine is distributed under an MIT licence. It runs on Docker stack which includes Nginx (latest version) and Gunicorn (version 22). It has been deployed on Linux Ubuntu 24.04 LTS, but should be compatible with other Linux distributions.

List of abbreviations

FAQ: Frequently Asked Questions; GLE: Galaxy Labs Engine; GTN: Galaxy Training Network; HTML: HyperText Markup Language; QC: Quality Control; SIG: Special Interest Group; SPOC: Single-cell and Spatial Omics Community; TSI: Threatened Species Initiative; UI: User Interface; VGP: Vertebrate Genomes Project.

Competing interests

The authors declare that they have no competing interests.

Funding

Galaxy Australia is supported by funding from the University of Melbourne, the Queensland Government , and the Australian BioCommons which is enabled by NCRIS via Bioplatforms Australia funding.

Authors' contributions

CH, AS, GP, WB were manuscript authors. CH developed the Labs Engine service. AS, JG, WM contributed to Labs Engine design. AS, WB, BB, PZ beta-tested the Labs Engine by using Galaxy Labs Engine to build and deploy Galaxy Labs across different Galaxy servers.

Acknowledgements

The authors would like to acknowledge Lab Creators whose feedback has been used to shape the design of Galaxy Labs and the Labs Engine, including Delphine Lariviere, Pavankumar Videm, and José Manuel Domínguez who co-designed the Single Cell Lab and Ove J.R. Gustafsson who created the Proteomics Lab.

Generative AI tools (ChatGPT 4o; June 2025) were used to revise and improve the clarity of the manuscript text. None of the manuscript content was generated entirely by AI.

References

1. The Galaxy platform for accessible, reproducible, and collaborative data analyses: 2024 update. Nucleic Acids Res. 2024;52(W1):W83-W94.
2. Hiltemann S, Rasche H, Gladman S, Hotz H-R, Larivière D, Blankenberg D, et al. Galaxy Training: A powerful framework for teaching!. PLoS Comput Biol. 2023;19(1):e1010752.
3. Rhie A, McCarthy SA, Fedrigo O, Damas J, Formenti G, Koren S, et al. Towards complete and error-free genome assemblies of all vertebrate species. Nature. 2021;592(7856):737-746.
4. Mehta S, Bernt M, Chambers M, Fahrner M, Föll MC, Gruening B, et al. A Galaxy of informatics resources for MS-based proteomics. Expert Rev Proteomics. 2023;20(11):251-266.
5. Afgan E, Sloggett C, Goonasekera N, Makunin I, Benson D, Crowe M, et al. Genomics virtual laboratory: a practical bioinformatics workbench for the cloud. PLoS One. 2015;10(10):e0140829. Available from: https://journals.plos.org/plosone/article/file?id=10.1371/journal.pone.0140829&type=printable
6. Libouban R, Mercier E, Chaussepied T, Batut B, Bretaudeau A, Le Corguillé G. Misconceptions about Galaxy debunked by the (French) Galaxy Community. In: jobim 2024; 2024. Available from: https://hal.science/hal-04694551/
7. Tekman M, Batut B, Ostrovsky A, Antoniewski C, Clements D, Ramirez F, et al. A single-cell RNA-sequencing training and analysis suite using the Galaxy framework. GigaScience. 2020;9(10):giaa102. Available from: https://academic.oup.com/gigascience/article-pdf/doi/10.1093/gigascience/giaa102/33956599/giaa102.pdf
8. Nasr E, Amato P, Bhardwaj A, Blankenberg D, Brites D, Cumbo F, et al. microGalaxy: A gateway to tools, workflows, and training for reproducible and FAIR analysis of microbial data. bioRxiv. 2024. Available from: https://www.biorxiv.org/content/10.1101/2024.12.23.629682.full.pdf

9. de Koning W, Miladi M, Hiltemann S, Heikema A, Hays JP, Flemming S, et al. NanoGalaxy: Nanopore long-read sequencing data analysis in Galaxy. GigaScience. 2020;9(10):giaa105. Available from: https://academic.oup.com/gigascience/article-pdf/9/10/giaa105/60688406/giaa105.pdf
10. Tekman M, Batut B, Ostrovsky A, Antoniewski C, Clements D, Ramirez F, et al. A single-cell RNA-sequencing training and analysis suite using the Galaxy framework. GigaScience. 2020;9(10):giaa102.
11. Moreno P, Huang N, Manning JR, Mohammed S, Solovyev A, Polanski K, et al. User-friendly, scalable tools and workflows for single-cell RNA-seq analysis. Nat Methods. 2021;18(4):327-328.
12. Django (2024) Django (version 5.1.4) https://github.com/django/django/releases/tag/5.1.4
13. Python (2024) Python (version 3.12). https://github.com/python/cpython/releases/tag/v3.12.11
14. Batut B, Bacon W, Zierep P, Bernt M, Soranzo N, Gustafsson OJR. Galaxy CoDex for finding tools, workflows, and training. In: GCC 2024-Galaxy Community Conference; 2024. https://doi.org/10.7490/f1000research.1119764.1

Web links and URLs

15. https://doi.org/10.25953/3eje-q240
16. https://doi.org/10.5281/zenodo.3967970
17. https://github.com/galaxyproject/galaxy_codex/blob/main/communities/genome/lab/
18. https://singlecell.usegalaxy.org
19. https://singlecell.usegalaxy.eu
20. https://singlecell.usegalaxy.org.au
21. https://microbiology.usegalaxy.org
22. https://microbiology.usegalaxy.eu
23. https://microbiology.usegalaxy.fr
24. https://microbiology.usegalaxy.org.au

Table 2: Feature catalogue for Galaxy Labs Engine

**Problem**	**Solution**
Each Galaxy server must be able to customize each Lab page with local context	Galaxy servers can specify a `<myserver>.yml` to override base configuration, enabling server-specific variables and content overrides
Each Galaxy server must be able to customize the introductory text, footer and stylesheets to meet local requirements	All Markdown snippets and static files can be customized by placing them in a `<HOSTNAME>` folder and specifying the custom path in `<myserver>.yml`.
Redundant content across different servers	Introduces variable interpolation across YAML and Markdown/HTML to reuse content dynamically

Lack of flexibility in content formatting	Supports Markdown and HTML, with popular UI libraries available for use. Markdown "snippets" are injected in three locations in the page where the Lab developer has complete creative freedom.
Cluttered interfaces with lack of consistency between Labs	All Labs are rendered from the same base template, which includes a standardized UI layout to ensure that Lab pages are intuitive and consistent. Dense content is structured into sections, tabs and collapsible elements.
Managing sections that are specific to certain Galaxy server(s)	Allows exclusion of specific items from certain hosts using the `exclude_from` directive the YAML content
Requirement to host content on GitHub creates a slow development cycle	Local rendering capability via a Python-based CLI to instantly preview changes before pushing to GitHub
Delay in Lab page updates due to server-side caching	Supports a `cache=false` flag to skip the caching. Local rendering bypasses cache entirely.
Capability for hosting static content (e.g. images) for use in Lab pages	Images can be placed in the "static" folder of the content repository and then referenced in Markdown and YAML content.
Lab developers need constructive error messages when the expected Lab structure/schema is violated	YAML content is validated and sanitized using the "pydantic" library. Error feedback is rendered into the requested web page for interpretation by the user.
A standardized, accessible mechanism for adding contributors	The content repository can include a CONTRIBUTORS file which lists GitHub usernames of Lab contributors. These are rendered at the bottom of the Lab page.
Ability to embed content from other websites into Lab pages	Since extended Markdown is supported, `iframe` elements can be used to embed content from any publicly visible website.
Links in the Lab page need to be screened to guarantee that the "Galaxy in Galaxy" anomaly does not occur.	All URLs in the rendered Lab page are programatically evaluated and configured to open in a new tab if they originate from the current web host.
Robust documentation for building new Lab pages	The GLE landing page provides interactive documentation for building a Galaxy Lab page
Landing on a new resource for the first time can be daunting for inexperienced users	GLE accepts a video_url attribute in the <server>.yml metadata, which enables a YouTube video to be easily embedded in the Introduction of the Lab.

Figure

Click here to access/download;Figure;fig-1.pdf

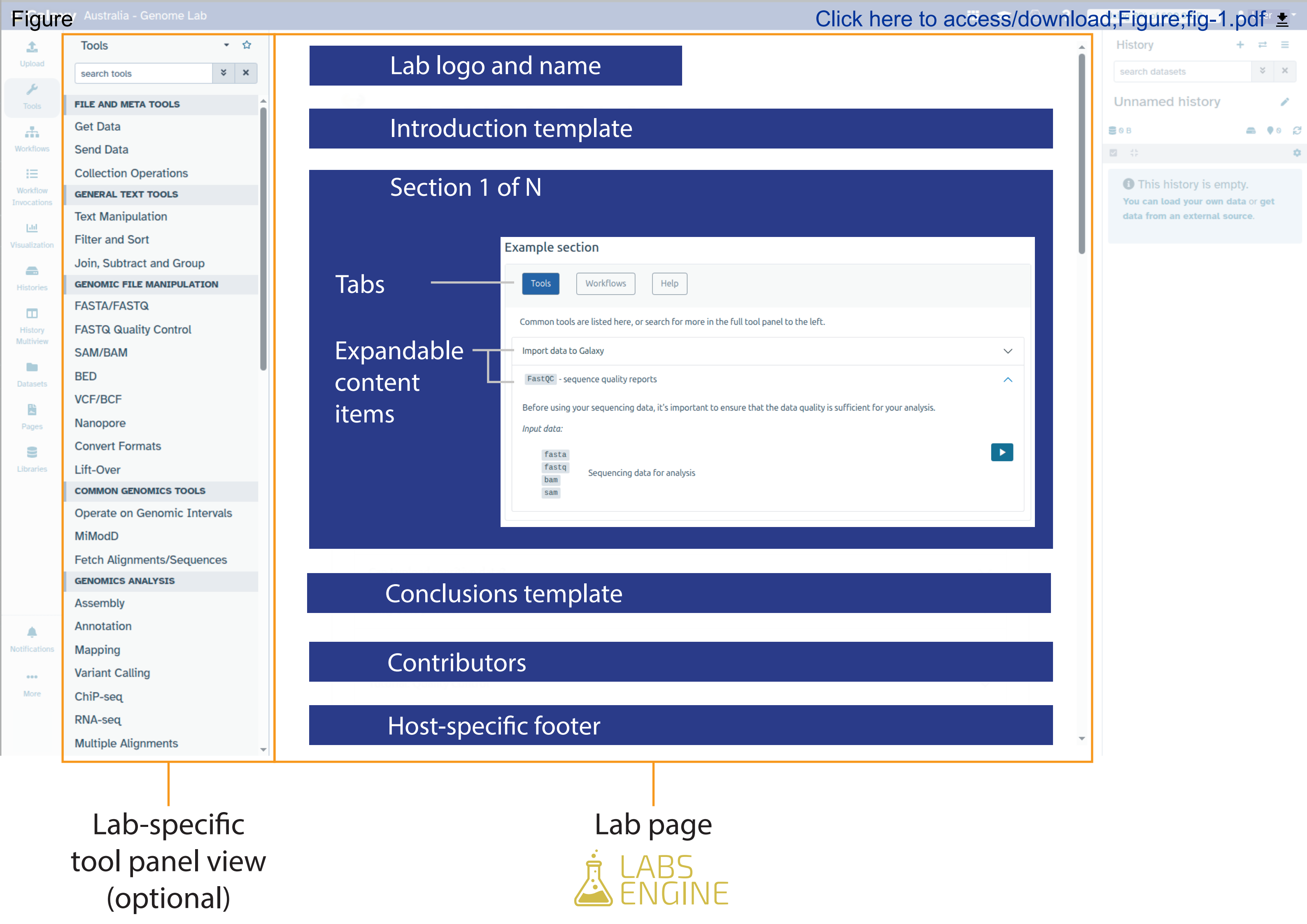

Figure Click here to access/download;Figure;fig-2.pdf

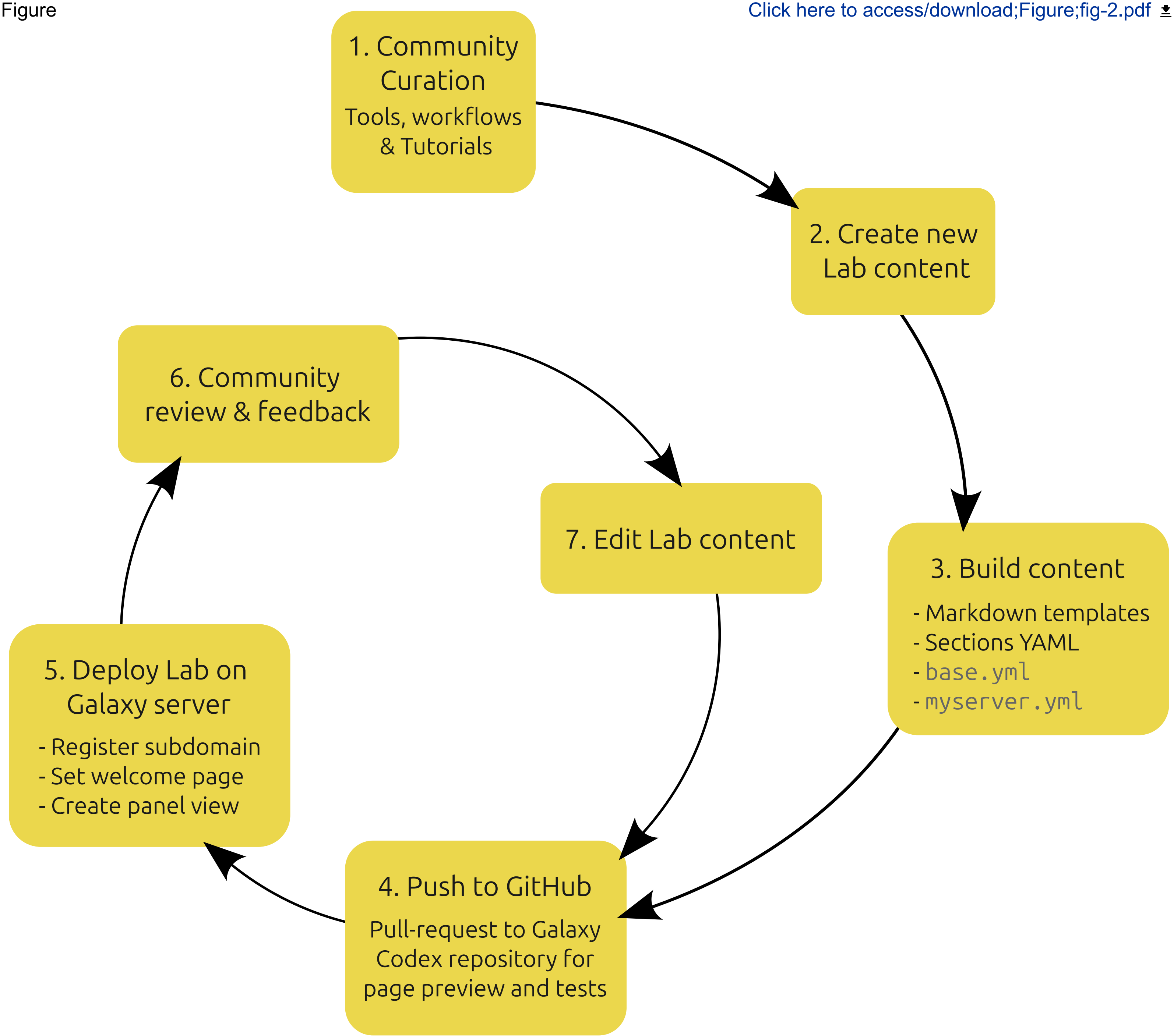

Figure

Click here to access/download;Figure;fig-3.pdf

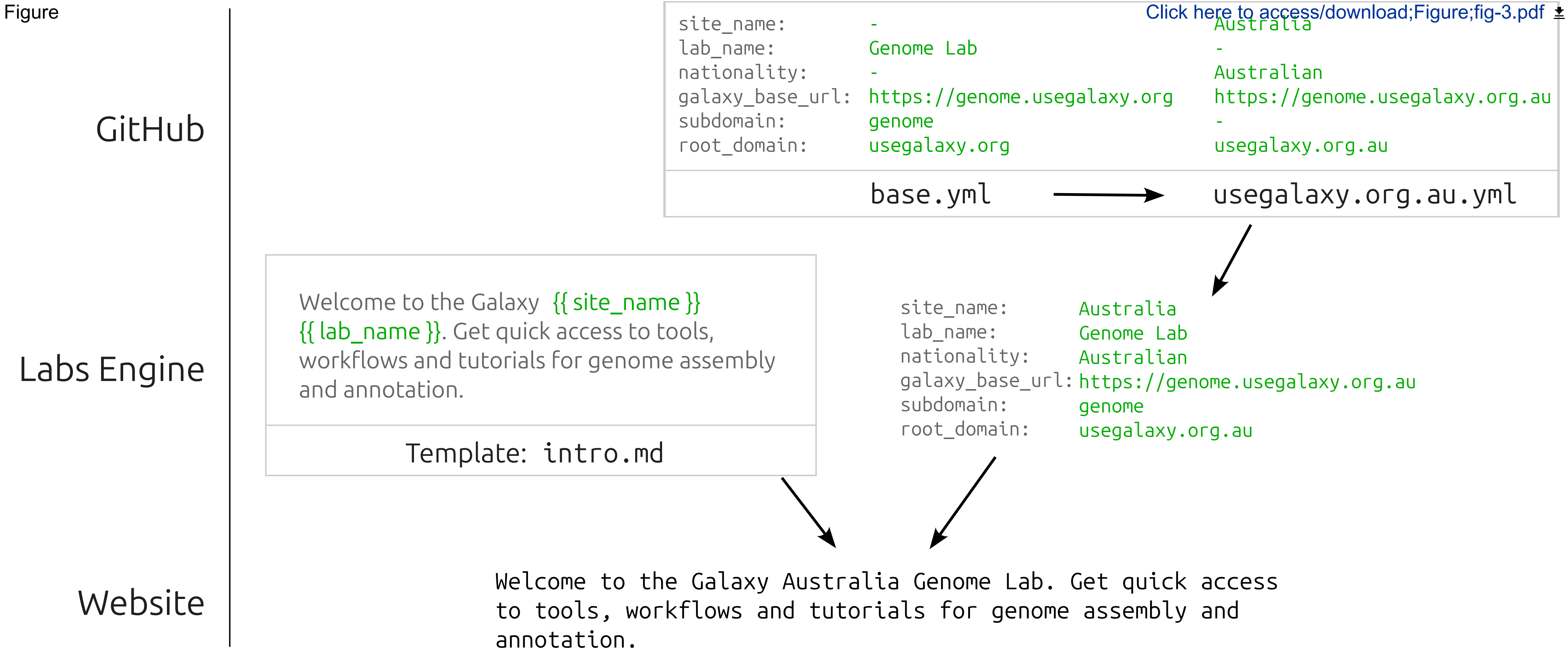

Figure

Click here to access/download;Figure;fig-4.pdf

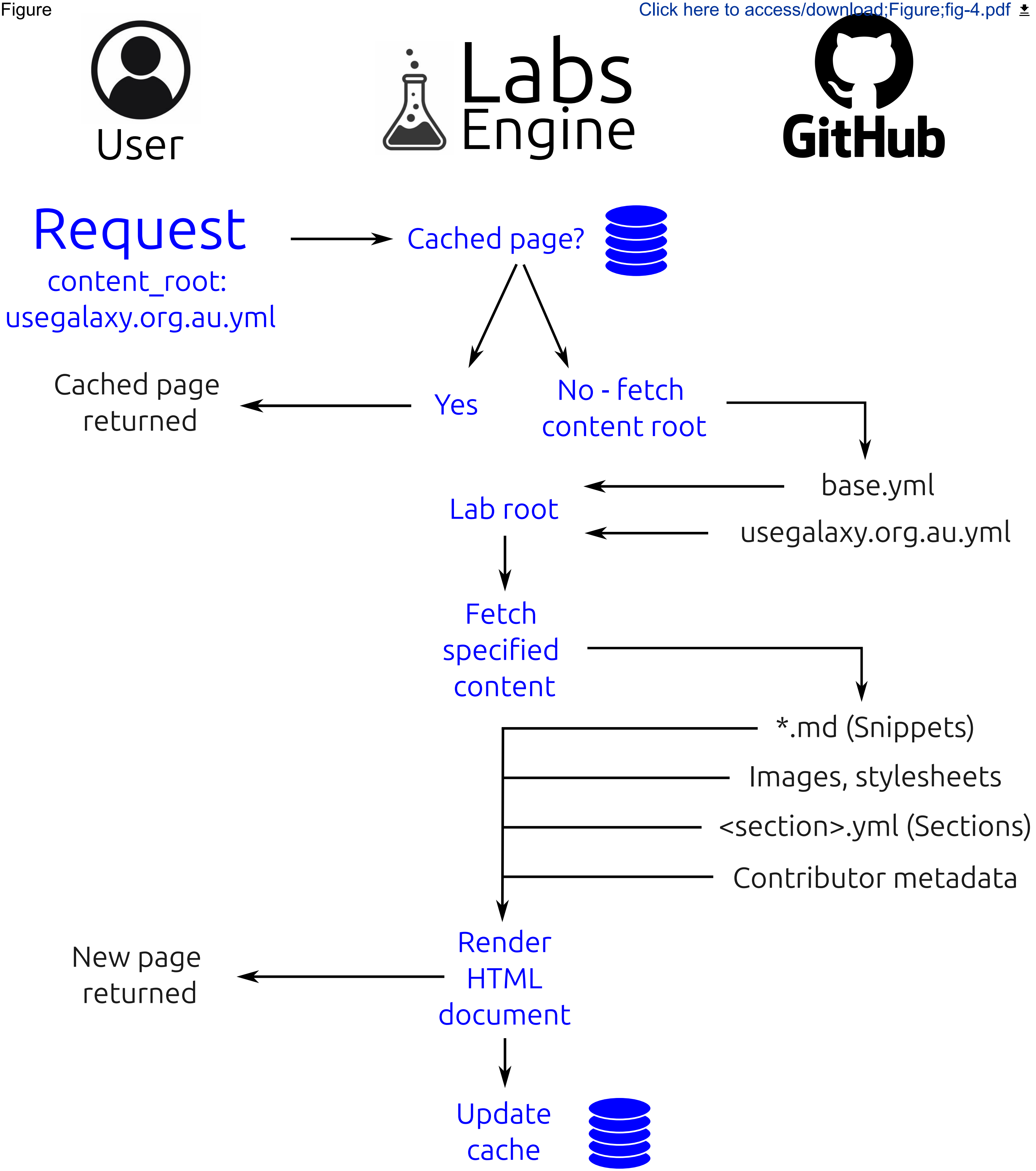

Cover letter Click here to access/download;Personal Cover;Cover letter.docx

Dr. Cameron Hyde
Data and Software Services, QCIF Ltd, Brisbane, Queensland, Australia

GigaScience
GigaScience Press, BGI Hong Kong Tech Co Ltd., 26F A Kings Wing Plaza 2, 1 On Kwan Street, Shek Mun, Sha Tin, NT, Hong Kong SAR

21 July 2025

Dear Editor,

We hope that you will take an interest in the manuscript we submit here, entitled “Community-curated Galaxy interfaces with the Galaxy Labs Engine”. The paper describes the vision, development and utility behind a new web service in the Galaxy Project ecosystem, which provides mechanisms to make the Galaxy web platform more approachable for researchers.

Galaxy is sometimes criticized for a user interface that is bloated with thousands of tools which are difficult to navigate - a paradigm which has emerged organically as the Galaxy ecosystem has expanded over decades. Our “Galaxy Labs”, facilitated by the software we present here, aim to overcome this by creating re-usable, extendable, community-curated “skins” over a Galaxy server. Galaxy Labs are a lens through which a user can interact with their local Galaxy, via an interface that caters to their research discipline. Rather than being faced with thousands of tools with arbitrary names, the user instead finds a curated panel of recommended tools, workflows and resources in a format that guides them through their data analysis journey.

We hope that that article will be an excellent fit with GigaScience, who have published many papers relating to the Galaxy Project in the past.

We would like to acknowledge that, while all information presented in the manuscript is the authors’ own, generative AI has been used to reword some sentences to improve flow and clarity. Since generative AI has not been used to generate complete sentences, we have not referenced it in the text as suggested in your guidelines, as we feel this would mislead the reader. We have instead described our use of generative AI in the Acknowledgements.

All authors have reviewed and accepted the final manuscript presented here, and we have no competing interests to declare. The manuscript is an original submission, and neither this manuscript or any manuscript presenting the Galaxy Labs Engine has been submitted to any other publisher.

Thank you for taking the time to review our manuscript.

Sincerely,

Dr. Cameron Hyde

Senior Software Developer
c.hyde@qcif.edu.au
