# Supplementary material for: Community-curated Galaxy interfaces with the Galaxy Labs Engine
Source: Gigascience. 2026 Apr 8;15:giag041. doi: 10.1093/gigascience/giag041 (PMC13148399; doi:10.1093/gigascience/giag041)
Supplement: giag041_Authors_Response_To_Reviewer_Comments_original_submission [file giag041_authors_response_to_reviewer_comments_original_submission.pdf]

Author's Response To Reviewer Comments

Please see the document of the review response. I have copied it here:

Reviewer#1
This paper reads well and describes an interesting contribution, the Galaxy Labs Engine (GLE) service. GLE may prove to be very useful to many users of Galaxy and perhaps of other workflow systems, too. There are numerous challenges faced by computational scientists not only in performing their work, but in sharing their artifacts for others to discover and reuse to perform their own work, too. GLE sounds promising in certain ways, especially (for my own interests) helping to streamline and standardize metadata about artifacts by generating boilerplate Lab content for users.

I would be very interested, as a reader, to see some discussion about how GLE is "fostering the FAIR principles", as mentioned in the abstract. There is no further discussion about FAIR after that, and I believe this is a missed opportunity by the authors to explain some of the advantages of GLE. Does GLE agree with the recently published FAIR principles for workflows? GLE helps organize and present metadata; does it also help with persistent identifiers? Does the metadata follow FAIR principles, too? Does using GLE help search engines, registries, and the like correctly interpret users' artifacts? The paper stands on its own as it is, but not discussing some of these ideas seems like a missed opportunity to frame GLE within the broader ecosystem (even beyond Galaxy) as a potentially forward-thinking design that can push innovation.

Some minor errors:
* Figure 1 is not referenced in the text.
* Page 4: "tenants" should be "tenets"

Finally, a potentially large error that should *definitely* be addressed: the "Author's Contributions" refer to a set of initials, "JG", which do not belong to any of the listed co-authors. This may indicate that a potential co-author has been omitted in error, and this absolutely should be addressed before publication!
Other coments: I am very concerned that the Author's Contributions refer to a set of initials ("JG") which do not match any co-author. This may indicate that co-authorship for the paper may need to change before publication due to an omission. I'm not sure if I should mark this paper as "major revision" for that one reason or not, but please take a look at this problem!
Response from authors:
1. We have incorporated a discussion of FAIR principles in p4-5
2. We assume they mean figure 4, as fig. 1 is referenced in many places. In any case, we have corrected the referencing and ordering of figures.
3. Corrected “tenants” to “tenets” as suggested
4. The author “JG” asked to be removed as they had not made a significant contribution to the work. We have removed the reference to the initals JG, which previously remained in error.
Reviewer#2
This manuscript presents a timely and technically sound contribution to the Galaxy community by introducing the Galaxy Labs Engine (GLE), a system designed to overcome the limitations of statically deployed "Galaxy Flavours". The work addresses a clear need for improved tool discoverability and community-driven customization in distributed bioinformatics platforms. The methodology is well described, and the examples provided demonstrate real-world applicability. The manuscript is suitable for publication after minor revisions as follows.

1. The website of Galaxy Labs Engine currently lacks a clear statement on how users or developers should cite this work/manuscript in "Cite this Lab".
2. References 7 and 10 are identical (both cite "Tekman et al., 2020, GigaScience, 9(10), giaa102"). The authors should remove the duplicate entry (e.g., Reference 10) and renumber subsequent.
3. In Table 2: Feature catalogue for Galaxy Labs Engine, some "Problem" or "Solution" entries end with a period (e.g., "Supports Markdown and HTML…"), while others do not (e.g., "Galaxy servers can specify…"). The punctuation style for all entries (either all with periods or all without) should be unified to improve table readability and formatting consistency.

4. There is no mention of Figure 4 in the main text, despite it appearing in the submitted materials. Either remove Figure 4 if it is not relevant to the narrative, or add a brief explanatory sentence in the appropriate section to reference and describe Figure 4.
5. The manuscript currently lacks Figure Legends, which should be provided.
Response from authors:
1. Excellent point, we will add a citation statement when this article has been published
2. Duplicate reference has been removed and references re-ordered accordingly
3. Corrected as suggested
4. Corrected as suggested, and figure references have been re-ordered accordingly
5. Figure legends have been added at the end of the article

Editorial Manager®

Cookie Preference Center

We use cookies which are necessary to make our site work. We may also use additional cookies to analyze, improve and personalize our content and your digital experience. For more information, see our Cookie Policy.

You may choose not to allow some types of cookies. However, blocking some types may impact your experience of our site and the services we are able to offer. See the different category headings below to find out more or change your settings.

Allow all

Manage Consent Preferences

Strictly Necessary Cookies

Always active

These cookies are necessary for the website to function and cannot be switched off in our systems. They are usually only set in response to actions made by you which amount to a request for services, such as setting your privacy preferences, logging in or filling in forms. You can set your browser to block or alert you about these cookies, but some parts of the site will not then work.

Functional Cookies

These cookies enable the website to provide enhanced functionality and personalisation. They may be set by us or by third party providers whose services we have added to our pages. If you do not allow these cookies then some or all of these services may not function properly.

Performance Cookies

These cookies allow us to count visits and traffic sources so we can measure and improve the performance of our site. They help us to know which pages are the most and least popular and see how visitors move around the site.

Targeting Cookies

These cookies may be set through our site by our advertising partners. They may be used by those companies to build a profile of your interests and show you relevant adverts on other sites. If you do not allow these cookies, you will experience less targeted advertising.

Cookie List

Clear

Apply Cancel

Consent Leg.Interest

Confirm my choices

Power
