# Supplementary material for: Community-curated Galaxy interfaces with the Galaxy Labs Engine
Source: Gigascience. 2026 Apr 8;15:giag041. doi: 10.1093/gigascience/giag041 (PMC13148399; doi:10.1093/gigascience/giag041)
Supplement: giag041_Reviewer_1_Report_original_submission — Reviewer 1 -- 8/25/2025 [file giag041_reviewer_1_report_original_submission.pdf]

Reviewer Report

Title: Community-curated Galaxy interfaces with the Galaxy Labs Engine

Version: Original Submission **Date:** 8/25/2025

Reviewer name: Reviewer 1

Reviewer Comments to Author:

This paper reads well and describes an interesting contribution, the Galaxy Labs Engine (GLE) service. GLE may prove to be very useful to many users of Galaxy and perhaps of other workflow systems, too. There are numerous challenges faced by computational scientists not only in performing their work, but in sharing their artifacts for others to discover and reuse to perform their own work, too. GLE sounds promising in certain ways, especially (for my own interests) helping to streamline and standardize metadata about artifacts by generating boilerplate Lab content for users.I would be very interested, as a reader, to see some discussion about how GLE is "fostering the FAIR principles", as mentioned in the abstract. There is no further discussion about FAIR after that, and I believe this is a missed opportunity by the authors to explain some of the advantages of GLE. Does GLE agree with the recently published FAIR principles for workflows? GLE helps organize and present metadata; does it also help with persistent identifiers? Does the metadata follow FAIR principles, too? Does using GLE help search engines, registries, and the like correctly interpret users' artifacts? The paper stands on its own as it is, but not discussing some of these ideas seems like a missed opportunity to frame GLE within the broader ecosystem (even beyond Galaxy) as a potentially forward-thinking design that can push innovation.Some minor errors:* Figure 1 is not referenced in the text.* Page 4: "tenants" should be "tenets"Finally, a potentially large error that should *definitely* be addressed: the "Author's Contributions" refer to a set of initials, "JG", which do not belong to any of the listed co-authors. This may indicate that a potential co-author has been omitted in error, and this absolutely should be addressed before publication!

Level of Interest

Please indicate how interesting you found the manuscript: Choose an item.

Quality of Written English

Please indicate the quality of language in the manuscript: Choose an item.

Declaration of Competing Interests

Please complete a declaration of competing interests, considering the following questions:

- Have you in the past five years received reimbursements, fees, funding, or salary from an organisation that may in any way gain or lose financially from the publication of this manuscript, either now or in the future?

- Do you hold any stocks or shares in an organisation that may in any way gain or lose financially from the publication of this manuscript, either now or in the future?
- Do you hold or are you currently applying for any patents relating to the content of the manuscript?
- Have you received reimbursements, fees, funding, or salary from an organization that holds or has applied for patents relating to the content of the manuscript?
- Do you have any other financial competing interests?
- Do you have any non-financial competing interests in relation to this paper?

If you can answer no to all of the above, write 'I declare that I have no competing interests' below. If your reply is yes to any, please give details below.

I declare that I have no competing interests.

I agree to the open peer review policy of the journal. I understand that my name will be included on my report to the authors and, if the manuscript is accepted for publication, my named report including any attachments I upload will be posted on the website along with the authors' responses. I agree for my report to be made available under an Open Access Creative Commons CC-BY license (http://creativecommons.org/licenses/by/4.0/). I understand that any comments which I do not wish to be included in my named report can be included as confidential comments to the editors, which will not be published.

Choose an item.

To further support our reviewers, we have joined with Publons, where you can gain additional credit to further highlight your hard work (see: https://publons.com/journal/530/gigascience). On publication of this paper, your review will be automatically added to Publons, you can then choose whether or not to claim your Publons credit. I understand this statement.

Yes Choose an item.
