# Supplementary material for: Community-curated Galaxy interfaces with the Galaxy Labs Engine
Source: Gigascience. 2026 Apr 8;15:giag041. doi: 10.1093/gigascience/giag041 (PMC13148399; doi:10.1093/gigascience/giag041)
Supplement: giag041_Reviewer_2_Report_original_submission — Reviewer 2 -- 2/3/2026 [file giag041_reviewer_2_report_original_submission.pdf]

Reviewer Report

Title: Community-curated Galaxy interfaces with the Galaxy Labs Engine

Version: Original Submission **Date:** 2/3/2026

Reviewer name: Reviewer 2

Reviewer Comments to Author:

This manuscript presents a timely and technically sound contribution to the Galaxy community by introducing the Galaxy Labs Engine (GLE), a system designed to overcome the limitations of statically deployed "Galaxy Flavours". The work addresses a clear need for improved tool discoverability and community-driven customization in distributed bioinformatics platforms. The methodology is well described, and the examples provided demonstrate real-world applicability. The manuscript is suitable for publication after minor revisions as follows.1. The website of Galaxy Labs Engine currently lacks a clear statement on how users or developers should cite this work/manuscript in "Cite this Lab".2. References 7 and 10 are identical (both cite "Tekman et al., 2020, GigaScience, 9(10), giaa102"). The authors should remove the duplicate entry (e.g., Reference 10) and renumber subsequent.3. In Table 2: Feature catalogue for Galaxy Labs Engine, some "Problem" or "Solution" entries end with a period (e.g., "Supports Markdown and HTML…"), while others do not (e.g., "Galaxy servers can specify…"). The punctuation style for all entries (either all with periods or all without) should be unified to improve table readability and formatting consistency.4. There is no mention of Figure 4 in the main text, despite it appearing in the submitted materials. Either remove Figure 4 if it is not relevant to the narrative, or add a brief explanatory sentence in the appropriate section to reference and describe Figure 4.5. The manuscript currently lacks Figure Legends, which should be provided.

Level of Interest

Please indicate how interesting you found the manuscript: Choose an item.

Quality of Written English

Please indicate the quality of language in the manuscript: Choose an item.

Declaration of Competing Interests

Please complete a declaration of competing interests, considering the following questions:

- Have you in the past five years received reimbursements, fees, funding, or salary from an organisation that may in any way gain or lose financially from the publication of this manuscript, either now or in the future?
- Do you hold any stocks or shares in an organisation that may in any way gain or lose financially from the publication of this manuscript, either now or in the future?

- Do you hold or are you currently applying for any patents relating to the content of the manuscript?
- Have you received reimbursements, fees, funding, or salary from an organization that holds or has applied for patents relating to the content of the manuscript?
- Do you have any other financial competing interests?
- Do you have any non-financial competing interests in relation to this paper?

If you can answer no to all of the above, write 'I declare that I have no competing interests' below. If your reply is yes to any, please give details below.

I declare that I have no competing interests

I agree to the open peer review policy of the journal. I understand that my name will be included on my report to the authors and, if the manuscript is accepted for publication, my named report including any attachments I upload will be posted on the website along with the authors' responses. I agree for my report to be made available under an Open Access Creative Commons CC-BY license (http://creativecommons.org/licenses/by/4.0/). I understand that any comments which I do not wish to be included in my named report can be included as confidential comments to the editors, which will not be published.

Choose an item.

To further support our reviewers, we have joined with Publons, where you can gain additional credit to further highlight your hard work (see: https://publons.com/journal/530/gigascience). On publication of this paper, your review will be automatically added to Publons, you can then choose whether or not to claim your Publons credit. I understand this statement.

Yes Choose an item.
